# Efficacy and safety of transarterial chemoembolization plus donafenib with or without camrelizumab for unresectable hepatocellular carcinoma: a propensity score matching analysis

**DOI:** 10.3389/fimmu.2026.1694973

**Published:** 2026-04-16

**Authors:** Qi Zhang, Zhenwu Lei, Hezi Qing, Xingwu Xie

**Affiliations:** 1 Department of Interventional Radiology, Renmin Hospital, Hubei University of Medicine, Shiyan, Hubei, China; 2 Department of Interventional Radiology, Qinghai University Affiliated Hospital, Xining, Qinghai, China; 3 Dongguan Community Health Service Center, Hanzhong, Shaanxi, China

**Keywords:** Camrelizumab, donafenib, interventional treatment, transarterial chemoembolization, unresectable hepatocellular carcinoma

## Abstract

**Purpose:**

To compare the efficacy and safety of transarterial chemoembolization plus donafenib (TACE+D) and TACE plus donafenib combined with camrelizumab (TACE+D+C) in unresectable hepatocellular carcinoma (uHCC), using propensity score matching (PSM) to minimize selection bias.

**Methods:**

A single-center retrospective study analyzed 278 patients with uHCC who received treatment between 2021 and 2024, and they were divided into TACE+D and TACE+D+C groups. PSM was used to perform 1:1 matching (58 patients per group). Tumor response, progression-free survival (PFS), overall survival (OS), and treatment-related adverse events (TRAEs) were compared; Cox regression was used to identify prognostic factors.

**Results:**

After PSM, 58 patients were included in each group. Compared with the TACE+D group, the TACE+D+C group demonstrated a significantly higher partial response (PR) (37.93% vs 20.69%, *P* = 0.041), objective response rate (ORR) (62.07% vs 36.21%, *P* = 0.005) and disease control rate (DCR) (86.21% vs 70.69%, *P* = 0.042). Notably, the TACE+D+C group achieved a remarkably longer mOS than the TACE+D group (23.1 months vs 12.0 months, *P* = 0.022). Similarly, median PFS was significantly prolonged in the TACE+D+C group compared with the TACE+D group (13.0 months vs 7.8 months, *P* = 0.007). Multivariable Cox regression identified Barcelona Clinic Liver Cancer (BCLC) stage (hazard ratio [HR] = 1.69, *P* = 0.029), Protein Induced by Vitamin K Absence or Antagonist-II (PIVKA-II) (HR = 3.66, *P* < 0.001), and alpha-fetoprotein (AFP) level (HR = 2.38, *P* < 0.001) as independent prognostic factors for OS. For PFS, independent prognostic factors were PIVKA-II (HR = 3.07, *P* < 0.001) and AFP level (HR = 2.52, *P* < 0.001). Although the TACE+D+C group exhibited immune-related adverse events (irAEs) (e.g., hypothyroidism and reactive cutaneous capillary endothelial proliferation), no significant differences were observed between the two groups in drug-related TRAEs, TRAEs after TACE, or Grade 3 TRAEs.

**Conclusion:**

The TACE+D+C group significantly improves ORR, PFS and OS in uHCC patients with a comparable safety profile to the TACE+D group, while the single-center retrospective design limits generalizability, warranting prospective studies.

## Introduction

Primary liver cancer is the sixth most common malignant tumor worldwide and the third leading cause of cancer-related mortality (1). Hepatocellular carcinoma (HCC) is the most common type of primary liver cancer, accounting for approximately 75 - 85% of all cases (2). Owing to the lack of early clinical symptoms in patients with HCC, most patients are diagnosed at advanced stages, which precludes the possibility of curative surgery. Even after resection, the 5-year recurrence rate approaches 70%, while the overall survival (OS) rate remains below 40% (3).

Transarterial chemoembolization (TACE) has been established as the primary treatment method for unresectable hepatocellular carcinoma (uHCC) according to international guidelines (EASL, ESMO, AASLD). Particularly for patients with stage B disease according to the Barcelona Clinic Liver Cancer (BCLC) classification, TACE is recommended as the first-line therapy (4–7). However, the objective response rate (ORR) of TACE alone generally ranges from 35% to 60%, and the median progression-free survival (mPFS) typically does not exceed 7–9 months. Furthermore, some patients may experience tumor recurrence or distant metastasis after TACE, and multiple TACE treatments may lead to deterioration of liver function (8). Consequently, strategies to prolong OS in patients following TACE are of critical clinical importance.

Molecularly targeted drugs and immune checkpoint inhibitors (ICIs) have garnered significant attention as emerging treatment modalities for uHCC (9). Compared with sorafenib, donafenib, a novel molecular targeted drug following sorafenib, has a longer median OS (mOS) and fewer adverse effects in a controlled, multicenter, randomized, head-to-head phase II-III trial (ZGDH3) (10). Currently, donafenib is the only first-line treatment drug demonstrated to be superior to sorafenib in Phase III clinical trials. It was approved in 2021 for use in patients with uHCC who have not previously received systemic therapy (11). However, the current research data on the combination of TACE and donafenib remain limited, particularly in terms of application studies involving triple therapy with ICIs. Camrelizumab is an immune checkpoint inhibitor. In the CARES-310 study, its combination with the molecular targeted drugs achieved a mOS of 23.8 months in the treatment of uHCC (12). Camrelizumab exerts its antitumor effects primarily by blocking the programmed cell death 1 (PD-1) and its ligand (PD-L1) signaling pathway, thereby reactivating the host immune system and enhancing tumoricidal activity (13).

Although camrelizumab currently has potential for improving oncological outcomes, to our knowledge, no studies to date have evaluated the efficacy of TACE combined with donafenib, with or without camrelizumab in treating uHCC. Previous studies did not employ propensity score matching (PSM), which may lead to selection bias and confounding factors. Therefore, we hypothesize that triple therapy (TACE+donafenib+camrelizumab, TACE+D+C) may improve outcomes versus dual therapy (TACE+donafenib, TACE+D). This real-world study employed PSM to compare the efficacy (tumor response, PFS, OS) and safety of these groups in uHCC. Our findings aim to clarify the clinical value of combined modalities in survival prolongation, tumor control, and adverse events (AEs) reduction, ultimately guiding personalized HCC management.

## Materials and methods

### Study design and patient selection

We collected clinical data from patients diagnosed with uHCC who received TACE combined with systemic therapy between January 2021 and January 2024 at the Department of Interventional Radiology, Affiliated Hospital of Qinghai University. Relevant clinical information, treatment-related adverse events (TRAEs), and survival data of the patients were documented. According to patients’ treatment preferences and clinical regimens, they were categorized into two cohorts: the TACE+D group and the TACE+D+C group. Serial follow-ups were conducted to monitor patients’ imaging findings and laboratory results, with detailed records of the occurrence of TRAEs and survival outcomes. This study was approved by the Ethics Committee of Affiliated Hospital of Qinghai University (Approval No: P-SL-2023-421) and performed in accordance with the Declaration of Helsinki and relevant ethical guidelines.

The inclusion criteria for this study were as follows: (1) patients who diagnosed with uHCC at BCLC stages B-C through imaging and laboratory examinations; (2) patients aged between 18 and 75 years; (3) patients with no clear indications for surgical treatment; (4) patients with preoperative liver function classified as Child-Pugh A/B; (5) patients with an Eastern Cooperative Oncology Group Performance Status (ECOG PS) of 0–1 before TACE; (6) patients with vascular invasion but without obvious extrahepatic metastasis; (7) patients not suitable for resection or local ablation, with a tumor burden less than 50% of the liver volume; (8) patients with at least one measurable lesion assessed in accordance with modified Response Evaluation Criteria in Solid Tumors (mRECIST) via CT or MRI; (9) patients with no prior relevant treatment; (10) patients without significant liver, kidney, or heart dysfunction.

The exclusion criteria for this study were as follows: (1) patients who had previously undergone surgery and experienced recurrence; (2) patients with secondary malignant tumors in the liver; (3) patients with complete obstruction of the portal vein accompanied by portal hypertension; (4) patients with a history of allergic reactions to contrast agents who were unable to undergo endovascular treatment; (5) patients with autoimmune diseases, or immunodeficiencies with severe immune dysfunction; (6) patients who were pregnant and patients with mental health disorders; (7) patients who underwent surgical treatment after TACE; (8) patients lost to follow-up; (9) patients who discontinued treatment with donafenib or camrelizumab due to intolerance to drug-related adverse reactions; (10) patients who developed liver failure after TACE; (11) patients who withdrew from follow-up and opted for an alternative treatment regimen; (12) patients were excluded from this study if their family members actively requested to withdraw from follow-up and terminate study treatment after the occurrence of AEs associated with either donafenib or camrelizumab.

### Methods

#### TACE procedure (14)

The patient was placed in a supine position, and the right inguinal region was prepared under sterile conditions. Local infiltration anesthesia was administered at the puncture site with 2% lidocaine, after which the modified Seldinger technique was employed to puncture the common femoral artery. A 5-Fr vascular sheath was then inserted into the vessel lumen. Under real-time fluoroscopic guidance, a Rosch Hepatic (RH) catheter was advanced through the vascular sheath and selectively positioned in either the celiac trunk or proper hepatic artery. Subsequent digital subtraction angiography (DSA) was performed to delineate critical tumor-related characteristics, including the anatomical distribution of tumor-feeding arteries (primary vessels and their branches), precise tumor localization, and tumor dimensions. A 2.7-Fr microcatheter was subsequently navigated to achieve superselective cannulation of the target tumor-supplying artery, through which a combination of chemotherapeutic agents (oxaliplatin 100 mg, epirubicin 40 mg, leucovorin calcium 0.2 g, and fluorouracil 0.75 g) was infused for transarterial chemoinfusion. Thereafter, a Lipiodol-epirubicin emulsion (containing epirubicin 20–40 mg) was injected via the same microcatheter, followed by sequential arterial embolization of the tumor’s blood supply until angiographic confirmation of complete occlusion of all feeding arteries. Finally, embolization was terminated when the predefined endpoint was achieved with polyvinyl alcohol (PVA) particles (350-560 μm) and gelatin sponge particles (560-710 μm), in strict adherence to the ‘Clinical Practice Guidelines for TACE Treatment of Hepatocellular Carcinoma in China’ (14). Postoperatively, the femoral artery puncture site was sealed with a vascular closure device and secured with compressive bandaging for hemostasis.

#### TACE materials and drugs

The intraoperative medications used were as follows: lidocaine (Hebei Tiancheng Pharmaceutical Co., Ltd.), calcium folinate (Jiangsu Hengrui Pharmaceutical Co., Ltd.), epirubicin (Zhejiang Haizheng Pharmaceutical Co., Ltd.), 5-fluorouracil (Jinyao Heping (Tianjin) Pharmaceutical Co., Ltd.), oxaliplatin (Nanjing Pharmaceutical Factory Co., Ltd.), lipiodol (Jiangsu Hengrui Pharmaceutical Co., Ltd.), and iodol injection (Jiangsu Hengrui Pharmaceutical Co., Ltd.).

The embolic materials used were polyvinyl alcohol (PVA) (Ailekang Pharmaceutical Co., Ltd.) and gelatin sponges (Ailekang Pharmaceutical Co., Ltd.).

#### Donafenib treatment regimen (11, 15)

Donafenib (Suzhou Zelgen Biopharmaceuticals Co., Ltd.) was initiated orally at 200 mg twice daily, 7 days after TACE completion. If patients experienced donafenib-related adverse reactions (e.g., hand-foot skin reaction, diarrhea, alopecia) without any camrelizumab-associated AEs, and the patients found the symptoms intolerable, the medication dose was reduced to 100 mg twice daily and appropriate symptomatic treatment was administered. If the patients tolerated the reduced dose and symptomatic treatment, the regimen of 100 mg twice daily was maintained. If the patients remained intolerant, donafenib was discontinued.

### Camrelizumab treatment regimen (13, 15)

Camrelizumab (Suzhou Suncadia Biopharmaceuticals Co., Ltd.) was administered intravenously at 200 mg 14 days after TACE. Patients were closely monitored for allergic reactions and immune-related AEs (irAEs). In the absence of these complications, subsequent immunotherapy cycles were repeated every 21 days. If patients developed camrelizumab-associated AEs without any donafenib-related adverse reactions, symptomatic treatment was administered first. Specifically, for reactive cutaneous capillary endothelial proliferation (RCCEP), topical glucocorticoid therapy was given; for hypothyroidism, levothyroxine treatment was initiated. If the patients achieved tolerance after symptomatic treatment, camrelizumab administration was continued. If intolerance persisted, camrelizumab was permanently discontinued.

### Serological index detection

Fasting venous blood samples (8-hour overnight fast) were collected from all patients at baseline before treatment initiation. Serum alpha-fetoprotein (AFP) was detected by chemiluminescent immunoassay (CLIA) using a Siemens Advia Centaur XP automatic chemiluminescence analyzer and matched reagent kits. Serum Protein Induced by Vitamin K Absence or Antagonist-II (PIVKA-II) was measured by CLIA with an Abbott Architect i2000SR automatic chemiluminescence immunoassay system and dedicated PIVKA-II detection kits. All detection procedures were performed in accordance with the standard operating procedures (SOPs) of the Central Clinical Laboratory of the Affiliated Hospital of Qinghai University (accredited by the national clinical laboratory quality control system). The unit of PIVKA-II was mAU/mL, a standardized quantitative unit calibrated against the internal reference standard of the detection kit, and the cut-off value of 400 mAU/mL was used for stratification analysis according to the Chinese Clinical Practice Guidelines for Hepatocellular Carcinoma (2024 version) (15).

### Follow-up

The first follow-up evaluation was conducted 3 weeks after TACE, including clinical assessment, triphasic dynamic contrast-enhanced abdominal organ imaging, and laboratory tests. A senior radiologist assessed efficacy based on mRECIST criteria (16), documented disease changes, and continuously monitored time to disease progression and time to death. If tumor progression was identified, repeat TACE was performed—this repeat TACE was defined as a disease progression event—after which all patients continued the original systemic treatment (with individual dose reduction only based on drug tolerance). In the absence of tumor progression, follow-up assessments were conducted every 3 months until the conclusion of the follow-up period. The cutoff date for data collection was July 1, 2025, and the occurrence of TRAEs was meticulously recorded.

### Evaluation of therapeutic efficiency

The tumor response was evaluated by a radiologist with over 10 years of experience using mRECIST criteria (16). Progressive disease (PD) was defined as an increase of ≥ 20% in the diameter of target lesions, as shown by CT, MRI, or ultrasound imaging, or the appearance of new lesions. A complete response (CR) was defined as the absence of any enhancement of lesions during the arterial phase, along with normal tumor markers. Partial response (PR) was defined as a ≥ 30% reduction in target lesion diameter during the arterial phase compared with previous measurements. Stable disease (SD) was defined as a tumor response that fails to meet the criteria for either PR or PD. The ORR was defined as the percentage of patients who had the best tumor response rating of CR or PR. Disease control rate (DCR) was defined as the percentage of patients who had the best tumor response rating of CR, PR, or SD.

### Observation parameters

#### Primary endpoints

PFS was defined as the time from the initiation of treatment to the observation of disease progression.

OS was defined as the time from treatment initiation until death for any reason.

#### Secondary endpoints

AEs related to TACE and medications (such as nausea, vomiting, and hand-foot skin reactions) were classified according to the Common Terminology Criteria for AEs Version 5.0 (CTCAE) (17).

### Variable selection for PSM

In a clinical study of patients with uHCC receiving TACE combined with systemic therapy, all relevant variables were selected as covariates in the PSM model, primarily based on the following considerations:

First, tumor-related characteristics (including tumor size, BCLC stage, portal vein invasion, and number of lesions) directly reflect the tumor’s biological behavior and its potential for invasion and metastasis. Higher tumor burden and more advanced stages are associated with poorer long-term survival rates. Second, hepatic reserve (including Child–Pugh class, albumin, aspartate aminotransferase [AST], etc.) determines treatment tolerability and serves as the foundation for long-term survival; more severe hepatic dysfunction is associated with a higher incidence of TRAEs and poorer prognosis. Patient performance status, as assessed by the ECOG PS score, is associated with treatment adherence and efficacy. A lower ECOG PS score indicates better treatment tolerance and a more favorable survival prognosis. Serum tumor markers (such as AFP and PIVKA-II) enable non-invasive monitoring of tumor progression and the risk of recurrence; combined testing further improves the accuracy of prognostic assessment (18, 19).

Therefore, the PSM analysis in this study was selected in accordance with clinical-pathological principles and clinical decision-making logic, and was used to effectively balance baseline confounding factors between groups. All of these associations have been validated in previous clinical studies (20, 21).

### Statistical analysis

Statistical analyses were performed using SPSS 26.0 (IBM, Armonk, NY, USA). Continuous variables were presented as mean ± standard deviation or median (range) for normally and non-normally distributed data, respectively. Categorical variables were expressed as counts (percentages). Between-group comparisons were conducted using Student’s t-test or Mann-Whitney U test for continuous variables, and the chi-squared test or Fisher’s exact test for categorical variables, as appropriate.

Survival outcomes, including PFS and OS, were estimated using the Kaplan-Meier method, with differences assessed using the log-rank test. Median survival times and their 95% confidence interval (CI) were calculated via the Brookmeyer-Crowley nonparametric method. Univariate and multivariate Cox regression analyses were employed to identify independent prognostic factors. Variables with a *P* value < 0.1 in univariate analysis were entered into the multivariate model. A two-sided *P* value < 0.05 was considered statistically significant. Kaplan-Meier curves were visualized using R software (Version 4.4.1). To address potential confounder imbalances between the two groups, PSM analysis was conducted via the one-to-one nearest neighbor method without replacement, with a caliper width of 0.02. Before and after PSM, quantitative data were presented as the mean ± standard deviation, frequency, or median with a 95% CI.

## Results

### Baseline patient characteristics

**Figure 1:**
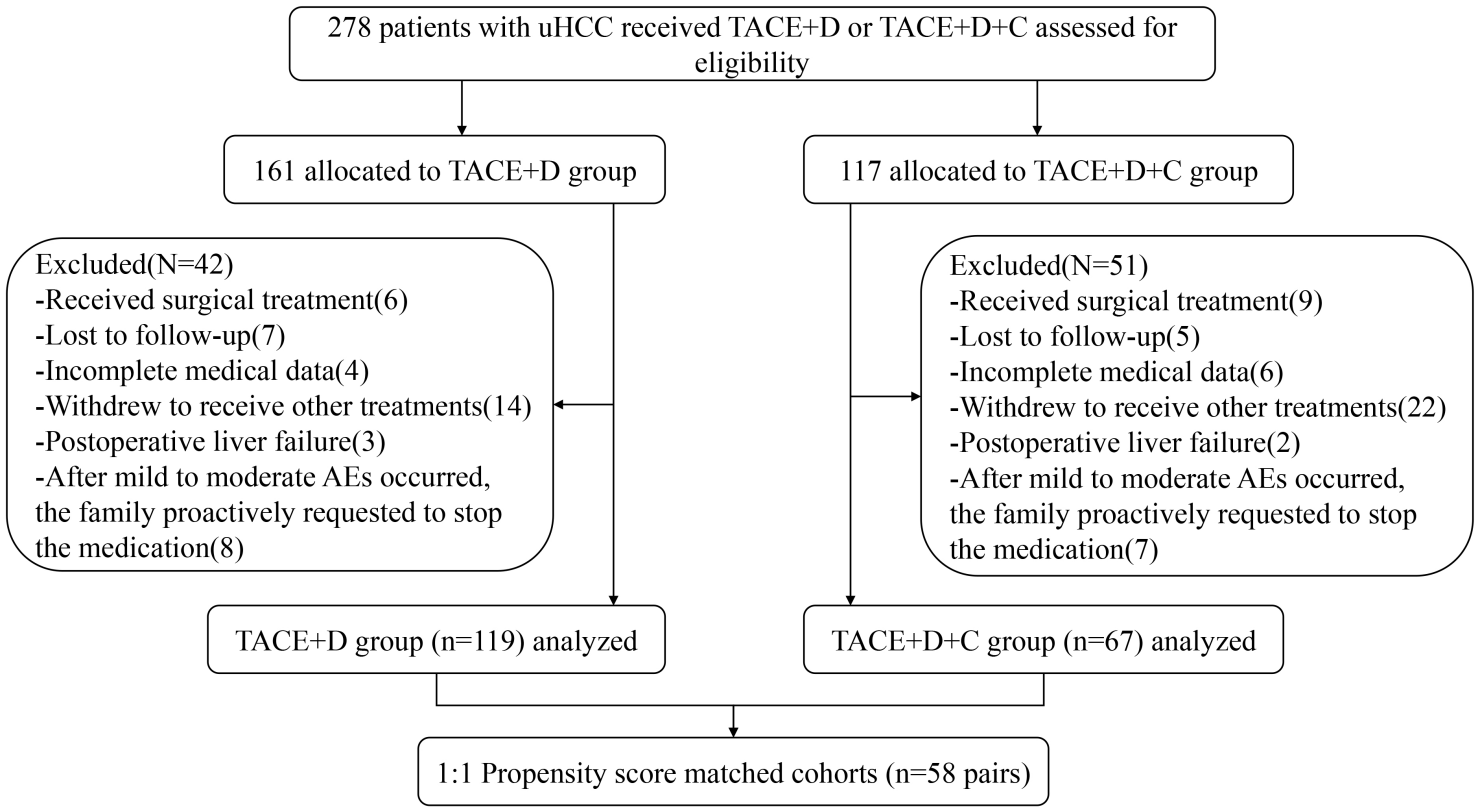
Patient flow chart. HCC, hepatocellular carcinoma; TACE+D, Transarterial Chemoembolization combined with donafenib; TACE+D+C, Transarterial Chemoembolization combined with donafenib and camrelizumab; AEs, Adverse events.

Under stringent inclusion and exclusion criteria, this study ultimately enrolled 119 patients in the TACE+D group and 67 in the TACE+D+C group (**Figure 1**). No significant differences (*P* > 0.05) were observed between the two groups in terms of age, sex, Child–Pugh class, hepatitis B virus (HBV), portal vein invasion, BCLC stage, albumin, AST, ECOG PS, PIVKA-II, AFP level, tumor size, number of lesions, or number of TACE procedures. After PSM, 58 patients from each group were analyzed. The baseline characteristics of the two groups after PSM were similar (*P* > 0.05) (**Table 1**).

**Table 1:** Baseline characteristics of patients in the two groups before and after PSM.

Characteristics	Before PSM		*P*-value	After PSM		*P*-value
	TACE+D Group (n=119) (%)	TACE+D+C Group (n=67) (%)		TACE+D Group (n=58) (%)	TACE+D+C Group (n=58) (%)	
Age(years)	57.34±10.09	55.42±9.98	0.795	55.16±9.49	56.38±10.22	0.505
<55	55 (46.2)	33 (49.3)	0.691	30 (51.7)	27 (46.5)	0.577
≥55	64 (53.8)	34 (50.7)		28(48.3)	31(53.5)	
Sex			0.611			0.305
Male	94 (78.99)	55 (82.09)		51 (87.93)	47 (81.03)	
Female	25 (21.01)	12 (17.91)		7 (12.07)	11 (18.97)	
Child-Pugh class			0.274			0.708
A	54 (45.38)	36 (53.73)		34 (58.62)	32 (55.17)	
B	65 (54.62)	31 (46.27)		24 (41.38)	26 (44.83)	
HBV			0.106			0.837
No	44 (36.97)	17 (25.37)		17 (29.31)	16 (26.59)	
yes	75 (63.03)	50 (74.63)		41 (70.69)	42 (72.41)	
Portal vein invasion			0.228			0.255
No	73 (61.34)	47 (70.15)		32 (55.17)	38 (65.52)	
Yes	46 (38.66)	20 (29.85)		26 (44.83)	20 (34.48)	
BCLC stage			0.319			0.258
B	73 (61.34)	46 (68.66)		31 (53.45)	37 (63.79)	
C	46 (38.66)	21 (31.34)		27 (46.55)	21 (36.21)	
Albumin(g/L)			0.594			0.560
<35	45 (37.82)	28 (41.79)		19 (32.76)	22 (37.93)	
≥35	74 (62.18)	39 (58.21)		39 (67.24)	36 (62.07)	
AST(U/L)			0.624			0.488
<25	7 (5.88)	6 (8.96)		3 (5.17)	6 (10.34)	
≥25	112 (94.12)	61 (91.04)		55 (94.83)	52 (89.66)	
ECOG PS			0.080			0.422
0	51 (42.86)	20 (29.85)		16 (27.59)	20 (34.48)	
1	68 (57.14)	47 (70.15)		42 (72.41)	38 (65.52)	
PIVKA-II (mAU/mL)			0.385			0.544
<400	43 (36.13)	20 (29.85)		16 (27.59)	19 (32.76)	
≥400	76 (63.87)	47 (70.15)		42 (72.41)	39 (67.24)	
AFP level (ng/mL)			0.362			0.255
<400	58 (48.74)	28 (41.79)		20 (34.48)	26 (44.83)	
≥400	61 (51.26)	39 (58.21)		38 (65.52)	32 (55.17)	
Tumor size(cm)			0.601			0.816
<5	27 (22.69)	13 (19.40)		11 (18.97)	12 (20.69)	
≥5	92 (77.31)	54 (80.60)		47 (81.03)	46 (79.31)	
Number of lesions			0.139			0.846
<3	87 (73.11)	42 (62.69)		38 (65.52)	37 (63.79)	
≥3	32 (26.89)	25 (37.31)		20 (34.48)	21 (36.21)	
Number of TACE			0.202			0.851
<2	62 (52.99)	42 (62.69)		33 (56.90)	34 (58.62)	
≥2	55 (47.01)	25 (37.31)		25 (43.10)	24 (41.38)	

Data are shown as the means ± standard deviations or as numbers (percentages).

BCLC stage, Barcelona Clinic Liver Cancer; AST, aspartate aminotransferase; ECOG PS, Eastern Cooperative Oncology Group performance status; PIVKA-II, Protein Induced by Vitamin K Absence or Antagonist-II; AFP, alpha-fetoprotein; TACE, Transarterial Chemoembolization; HBV, Hepatitis B Virus; PSM, propensity score matching.

### Sensitivity analysis

To further verify the robustness of the survival benefit associated with TACE+D+C compared with TACE+D in patients with uHCC, we conducted a stratified sensitivity analysis by adjusting for different subsets of confounding covariates. We performed unadjusted and full-covariate adjusted baseline analyses, with TACE+D as the reference group. For OS, the unadjusted HR for the TACE+D+C group was 0.65 (95% CI: 0.45-0.94, *P* = 0.025), and the fully adjusted HR (adjusted for age, sex, Child-Pugh class, HBV, portal vein invasion, BCLC stage, albumin level, AST, ECOG PS, PIVKA-II, AFP level, tumor size, number of lesions, and number of TACE procedures) was 0.35 (95% CI: 0.22-0.54, *P* < 0.001). For PFS, the unadjusted HR for the TACE+D+C group was 0.57 (95% CI: 0.39-0.83, *P* = 0.003), and the fully adjusted HR was 0.34 (95% CI: 0.21-0.53, *P* < 0.001) (**Table 2**).

**Table 2:** Sensitivity Analysis of OS and PFS for TACE+D+C group vs. TACE+D group (TACE+D as reference group).

Time	Group	Unadjusted HR (95%CI)	*P*-value	Adjusted* HR (95%CI)	*P*-value
OS	TACE+D ^a^				
OS	TACE+D+C	0.65 (0.45-0.94)	0.025	0.35 (0.22-0.54)	<.001
PFS	TACE+D ^a^				
PFS	TACE+D+C	0.57 (0.39-0.83)	0.003	0.34 (0.21-0.53)	<.001

*Full covariates: Age, sex, Child-Pugh class, HBV, portal vein invasion, BCLC stage, albumin, AST, ECOG PS, PIVKA-II, AFP level, tumor size, number of lesions, number of TACE.

**^a^**As a reference group for adjusting covariates. HR, hazard ratio; CI, confidence interval; OS, overall survival; PFS, Progression-free survival; TACE+D, Transarterial Chemoembolization combined with donafenib; TACE+D+C, Transarterial Chemoembolization combined with donafenib and camrelizumab; PSM, propensity score matching.

### Treatment assignment prediction model

Logistic regression was used with treatment as the dependent variable and all PSM covariates as independent variables. The model showed good performance with an area under the curve (AUC) of 0.726 (95% CI: 0.65-0.79), indicating that the selected covariates could effectively discriminate patients who received different treatments (**Supplementary Figure 1**).

### Rosenbaum analysis

To assess the robustness of PSM results to unmeasured confounding, we performed Rosenbaum sensitivity analysis for paired survival data following the original statistical procedure (22) with adaptations for oncology prognostic research involving PSM survival data (23). A stratified log-rank test (stratified by PSM match ID) was first conducted to obtain the chi-square statistic of the treatment effect on survival outcome. We then manually calculated the upper bound *P*-values for different gamma values (1.0 to 2.0 with 0.1 increments) using the core Rosenbaum formula. The results showed that the observed treatment effect remained statistically significant at a gamma value of 1.6 (upper limit *P*-value = 0.049), indicating that an unobserved confounder would need to increase the odds of treatment assignment by at least 1.6-fold to nullify the significant association between treatment and survival. This confirms the robustness of our findings to potential unmeasured confounding factors (**Supplementary Table 1**).

### Treatment outcomes

#### Evaluation of tumor response after treatment in the two groups

**Figure 2:**
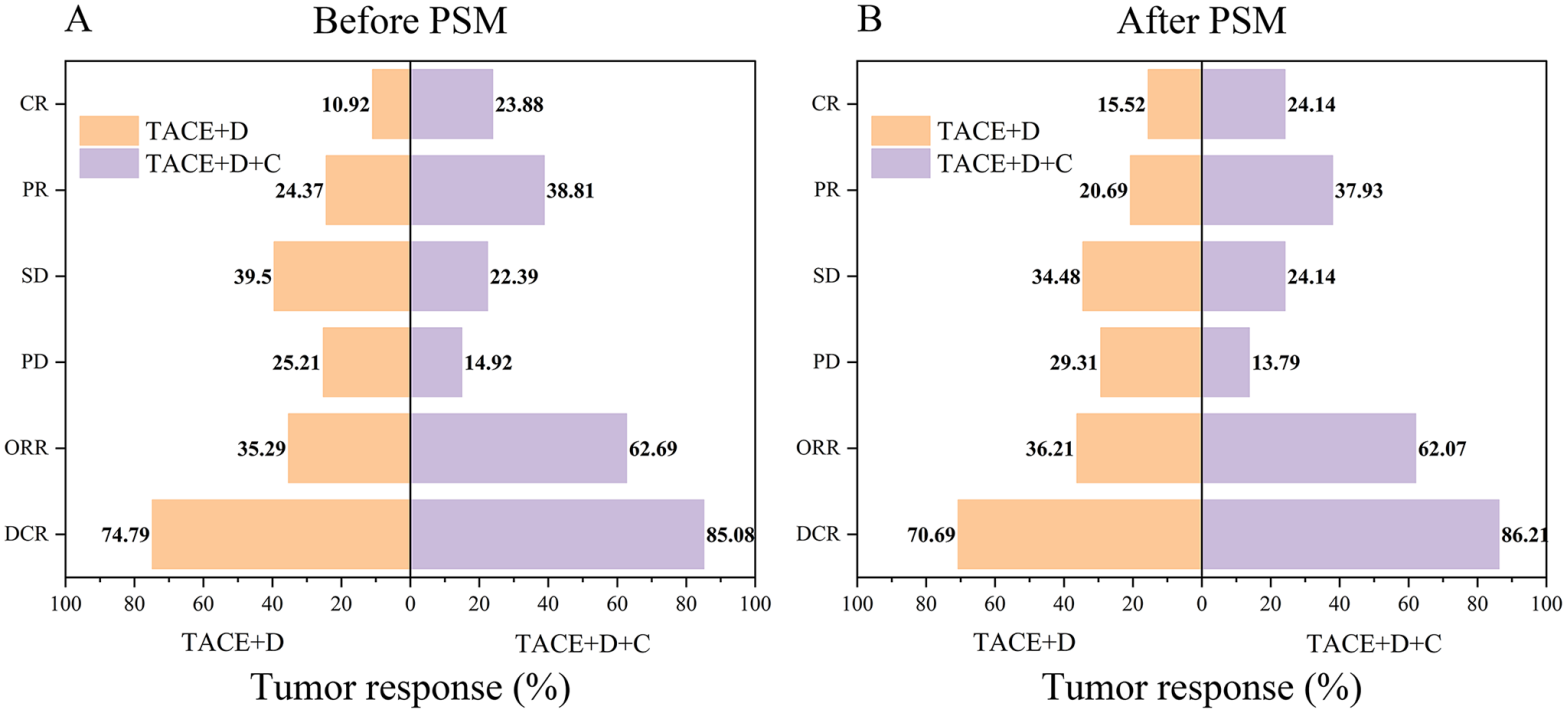
Treatment responses before PSM **(A)** and after PSM **(B)** for the two groups. Data are presented as numbers (percentages). TACE+D, Transarterial Chemoembolization combined with donafenib; TACE+D+C, Transarterial Chemoembolization combined with donafenib and camrelizumab; CR, complete response; PR, partial response; SD, stable disease; PD, progressive disease; ORR, objective response rate; DCR, disease control rate; PSM, propensity score matching.

The tumor response assessment was conducted according to the mRECIST (**Figure 2**). Before PSM, there were statistically significant differences between the TACE+D group and the TACE+D+C group in terms of CR (10.92% vs 23.88%, *P* = 0.019), PR (24.37% vs 38.81%, *P* = 0.038), SD (39.50% vs 22.39%, *P* = 0.017), and ORR (35.29% vs 62.69%, *P* < 0.001). However, no statistically significant differences were found between the TACE+D group and the TACE+D+C group in terms of PD (25.21% vs 14.92%, *P* = 0.101) or DCR (74.79% vs 85.08%, *P* = 0.101) (**Figure 2A**; **Supplementary Table 2**).

After PSM, the TACE+D+C group was significantly different from the TACE+D group in terms of PR (37.93% vs 20.69%, *P* = 0.041), PD (13.79% vs 29.31%, *P* = 0.042), ORR (62.07% vs 36.21%, *P* = 0.005), and DCR (86.21% vs 70.69%, *P* = 0.042). However, there were no significant differences in CR (24.14% vs 15.52%, *P* = 0.244) and SD (24.14% vs 34.48%, *P* = 0.221) (**Figure 2B**; **Supplementary Table 2**).

#### Survival analysis

With a follow-up cutoff date of July 1, 2025, complete follow-up data were obtained for all 186 enrolled patients. The maximum follow-up duration was 48 months. The entire study cohort (n=186, including 119 patients in the TACE+D group and 67 patients in the TACE+D+C group) had a mOS of 17.3 months (95% CI: 11.66 months-22.93 months). After PSM, 20 of 58 patients (34.48%) in the TACE+D+C group were censored during follow-up, compared with 15 of 58 patients (25.86%) in the TACE+D group.

**Figure 3:**
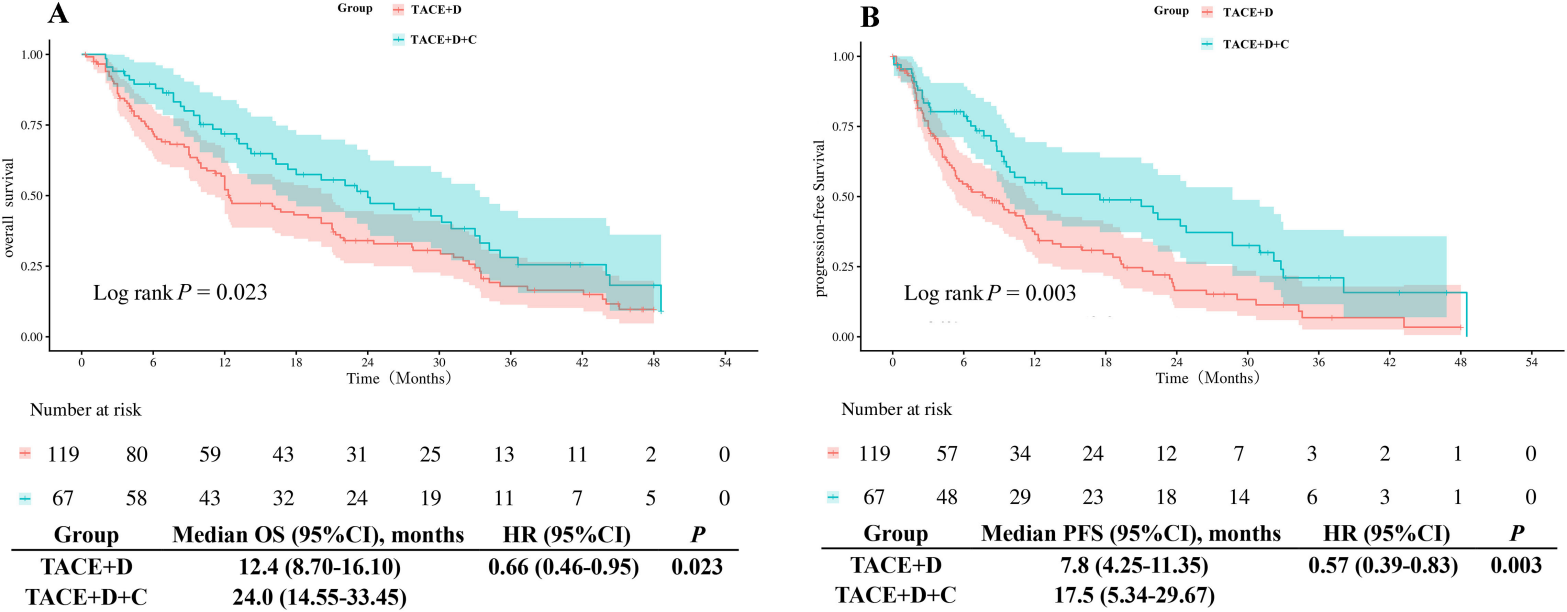
Kaplan-Meier analyses of OS **(A)** and PFS **(B)** according to the two groups before PSM. TACE+D, Transarterial Chemoembolization combined with donafenib; TACE+D+C, Transarterial Chemoembolization combined with donafenib and camrelizumab; OS, overall survival; PFS, progression-free survival; HR, hazard ratio; CI, confidence interval.

Before PSM, the mOS in the TACE+D group was 12.4 months (95% CI: 8.70 months-16.10 months), which was shorter than that in the TACE+D+C group (24.0 months; 95% CI: 14.55 months-33.45 months; *P* = 0.023) (**Figure 3A**). Similarly, the mPFS in the TACE+D group was 7.8 months (95% CI: 4.25 months-11.35 months), which was significantly shorter than the 17.5 months (95% CI: 5.34 months-29.67 months) (*P* = 0.003) observed in the TACE+D+C group. (**Figure 3B**).

**Figure 4:**
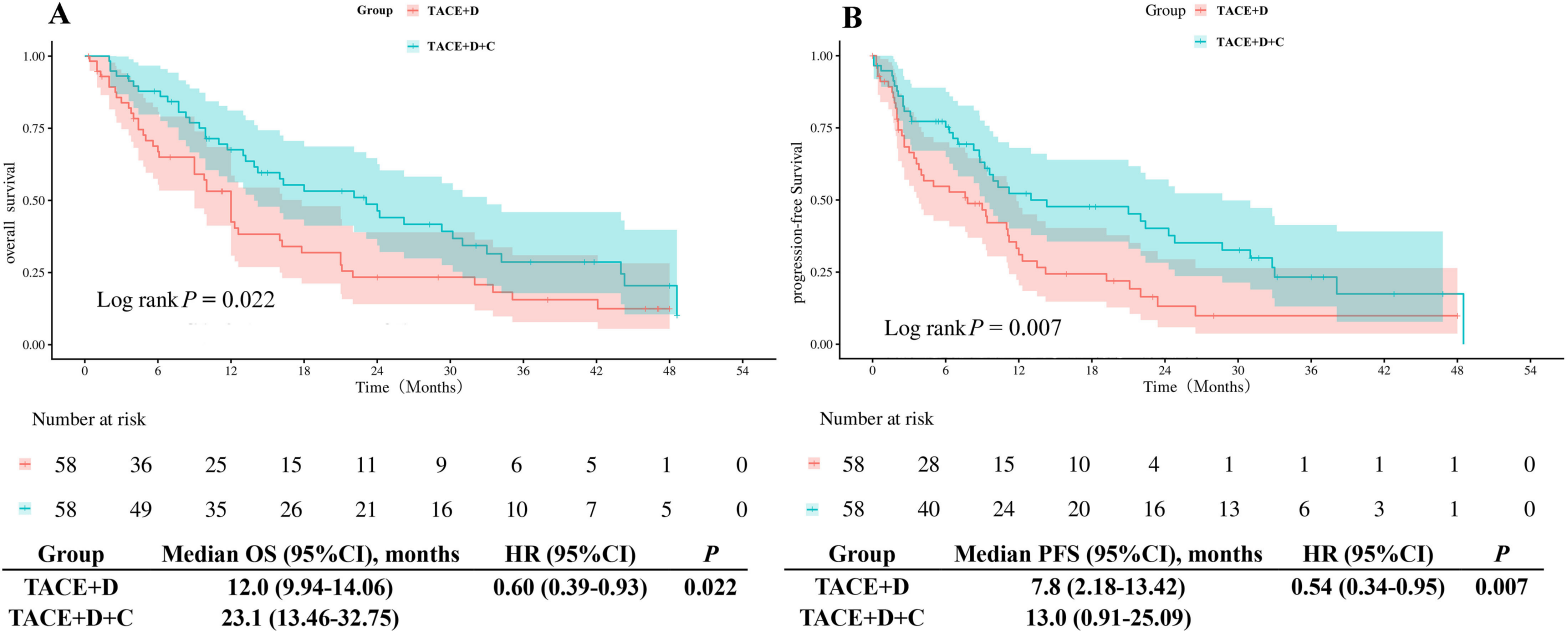
Kaplan-Meier analyses of OS **(A)** and PFS **(B)** according to the two groups after PSM. TACE+D, Transarterial Chemoembolization combined with donafenib; TACE+D+C, Transarterial Chemoembolization combined with donafenib and camrelizumab; OS, overall survival; PFS, progression-free survival; HR, hazard ratio; CI, confidence interval.

After PSM, the mOS in the TACE+D group was 12.0 months (95% CI: 9.94 months-14.06 months), which was significantly shorter than that in the TACE+D+C group (23.1 months; 95% CI: 13.46 months-32.75 months; *P* = 0.022) (**Figure 4A**). Similarly, the mPFS in the TACE+D group was 7.8 months (95% CI: 2.18 months-13.42 months), whereas it was 13.0 months (95% CI: 0.91 months-25.09 months; *P* = 0.007) in the TACE+D+C group (**Figure 4B**).

#### Milestone analysis

To further validate the temporal stability of treatment efficacy and minimize time-related bias, we conducted milestone analyses at 6- and 12-months post-treatment initiation. Inclusion criteria required patients to remain progression-free and alive at the respective milestone time points.

After PSM matching, 85 patients (36 in the TACE+D group, 49 in the TACE+D+C group) met the 6-month milestone criteria for OS analysis; 67 patients (27 in the TACE+D group, 40 in the TACE+D+C group) met the 6-month milestone criteria for PFS analysis; 60 patients (25 in the TACE+D group, 35 in the TACE+D+C group) met the 12-month milestone criteria for OS analysis; 39 patients (15 in the TACE+D group, 24 in the TACE+D+C group) met the 12-month milestone criteria for PFS analysis.

**Figure 5:**
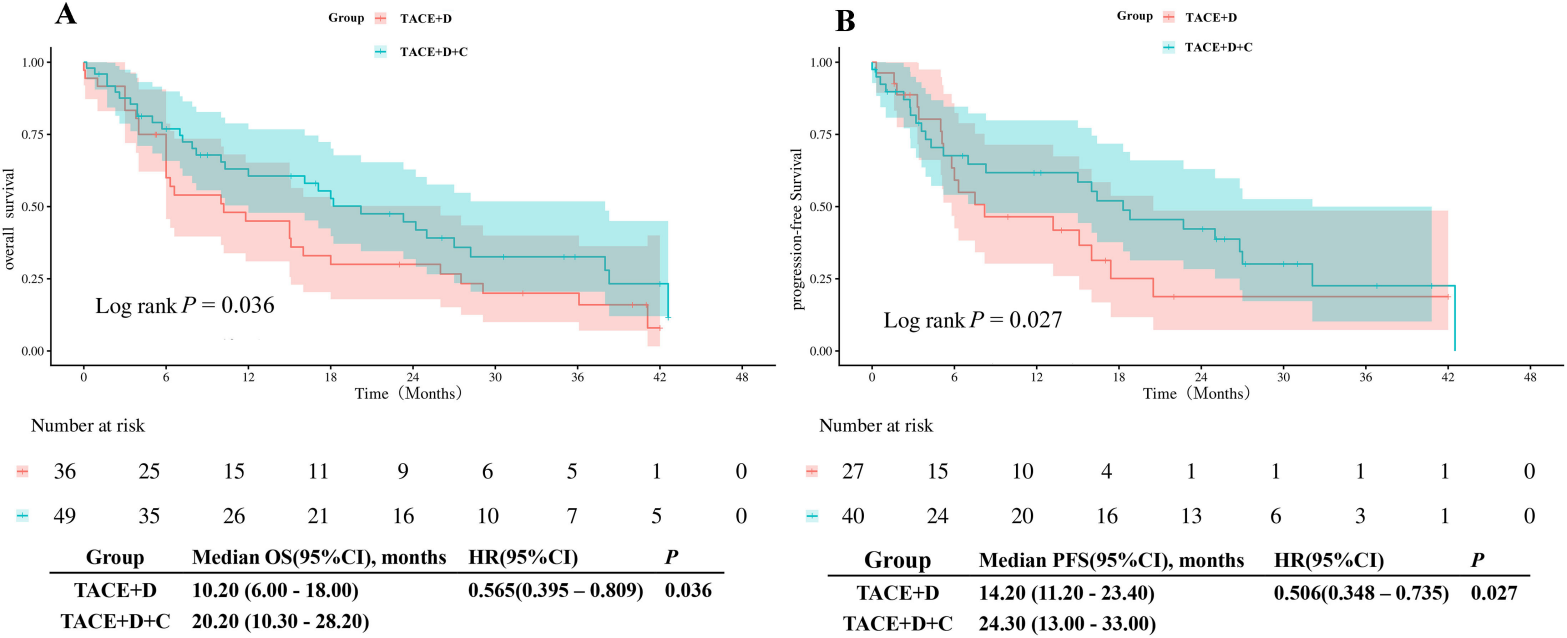
The 6-month milestone analysis for OS and PFS is presented. Figure **(A)** shows the OS curve, while Figure **(B)** depicts the PFS curve. TACE+D, Transarterial Chemoembolization combined with donafenib; TACE+D+C, Transarterial Chemoembolization combined with donafenib and camrelizumab; OS, overall survival; PFS, progression-free survival; HR, hazard ratio; CI, confidence interval.

Six-month milestone analysis revealed that post-milestone mOS was 20.20 months (95% CI: 10.30 months-28.20 months) in the TACE+D+C group versus 10.20 months (95% CI: 6.00 months-18.00 months) in the TACE+D group (*P* = 0.036; **Figure 5A**). For post-milestone mPFS, the TACE+D+C group achieved 24.30 months (95% CI: 13.00 months-33.00 months) compared with 14.20 months in the TACE+D group (95% CI: 11.20 months-23.40 months; *P* = 0.027; **Figure 5B**).

**Figure 6:**
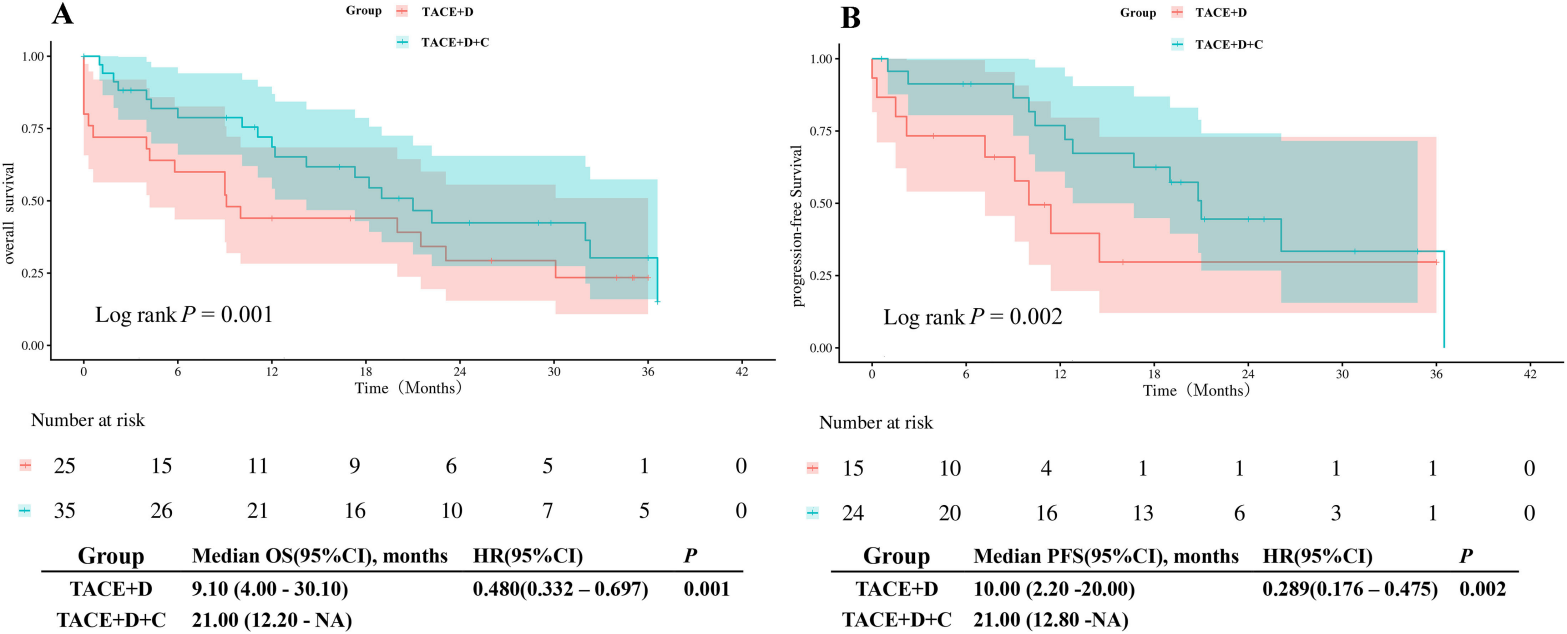
The 12-month analysis milestone for OS and PFS is presented, with Figure **(A)** showing the OS curve and Figure **(B)** depicting the PFS curve. TACE+D, Transarterial Chemoembolization combined with donafenib; TACE+D+C, Transarterial Chemoembolization combined with donafenib and camrelizumab; OS, overall survival; PFS, progression-free survival; HR, hazard ratio; CI, confidence interval.

For the 12-month landmark analysis, post-milestone mOS was 21.00 months (95% CI: 12.20 months-NA) in the TACE+D+C group, compared with 9.10 months (95% CI: 4.00 months-30.10 months) in the TACE+D group (*P* = 0.001; **Figure 6A**). Similarly, post-milestone mPFS in the TACE+D+C group was 21.00 months (95% CI: 12.80 months-NA), versus 10.00 months (95% CI: 2.20 months-20.00 months) in the TACE+D group (*P* = 0.002; **Figure 6B**).

#### Analysis of prognostic factors

**Figure 7:**
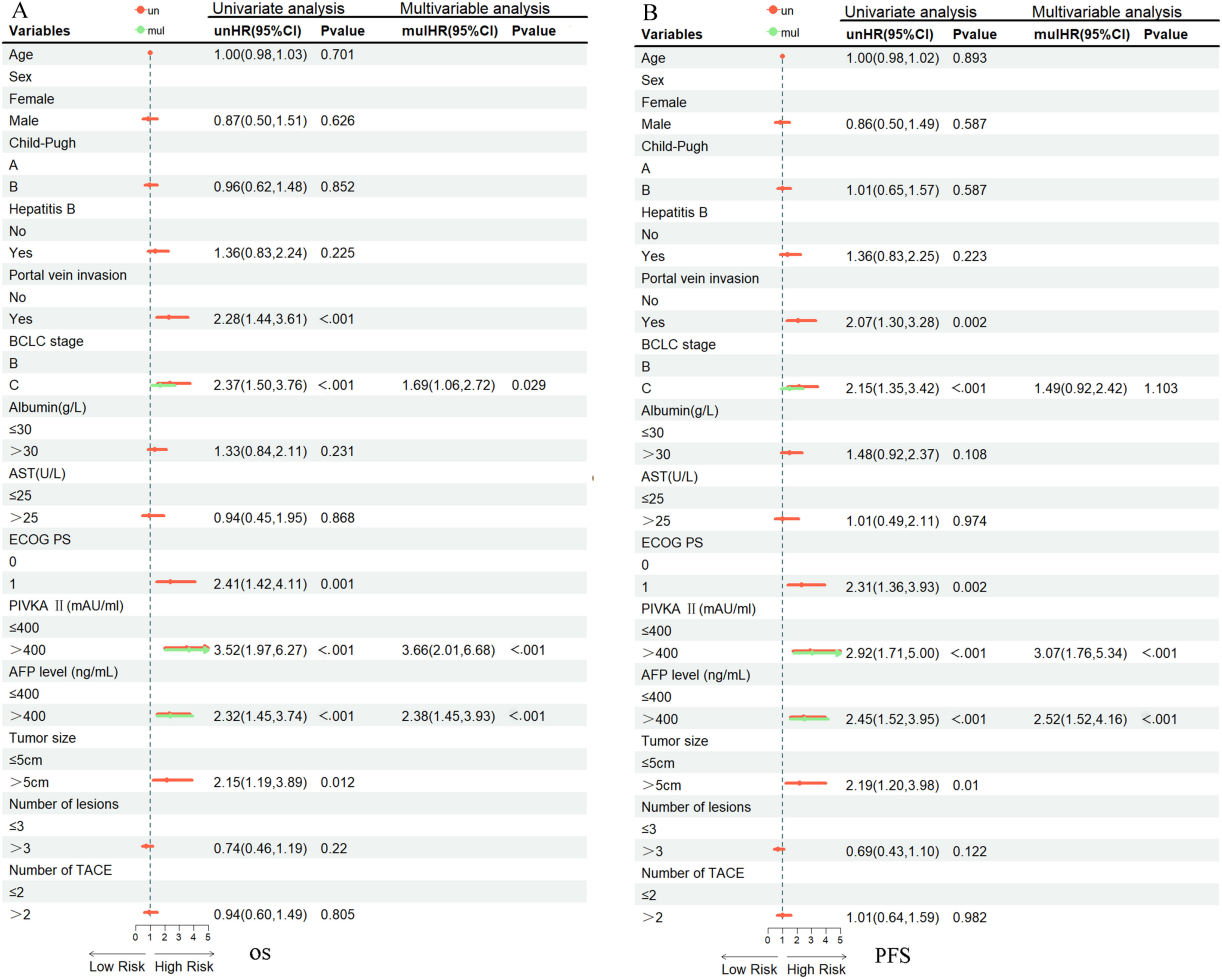
Shows univariate and multivariate predictors of PFS and OS after PSM. The data are presented as HR (95% CI). **(A)** shows the univariate and multivariate analyses associated with OS. **(B)** presents the univariate and multivariate analyses related to PFS. Un, univariate; Mul, multivariate; BCLC, Barcelona Clinic Liver Cancer; PIVKA-II, Protein Induced by Vitamin K Absence or Antagonist-II; AFP, alpha-fetoprotein; ECOG PS, Eastern Cooperative Oncology Group performance status; AST, aspartate aminotransferase; TACE, Transarterial Chemoembolization; HR, hazard ratio; CI, confidence interval.

After PSM, the univariate and multivariate analyses of the matched cohorts were illustrated in **Figure 7**. The Cox regression analysis demonstrated that BCLC stage (B vs C; HR = 1.69, 95% CI: 1.06-2.72, *P* = 0.029), PIVKA-II level (≤ 400 vs > 400 mAU/mL; HR = 3.66, 95% CI: 2.01-6.68, *P* < 0.001), and AFP level (≤ 400 vs > 400 ng/mL; HR = 2.38, 95% CI: 1.45-3.93, *P* < 0.001) were independent prognostic factors for OS (**Figure 7A**). Multivariate analysis showed that PIVKA-II (≤ 400 vs > 400 mAU/mL) (HR = 3.07, 95% CI: 1.76-5.34, *P* < 0.001) and AFP (≤ 400 vs > 400 ng/mL) (HR = 2.52, 95% CI: 1.52-4.16, *P* < 0.001) were independent prognostic factors for PFS (**Figure 7B**).

### Subgroup analysis

Based on the results of multivariate Cox regression analysis, we compared the differences in OS and PFS between the TACE+D+C and TACE+D groups across subgroups stratified by AFP level, PIVKA-II, and BCLC stage using Kaplan-Meier survival curves and the log-rank test. This analysis was performed to evaluate whether treatment efficacy differed significantly across these distinct subgroups.

#### Subgroup analysis stratified by AFP level

**Figure 8:**
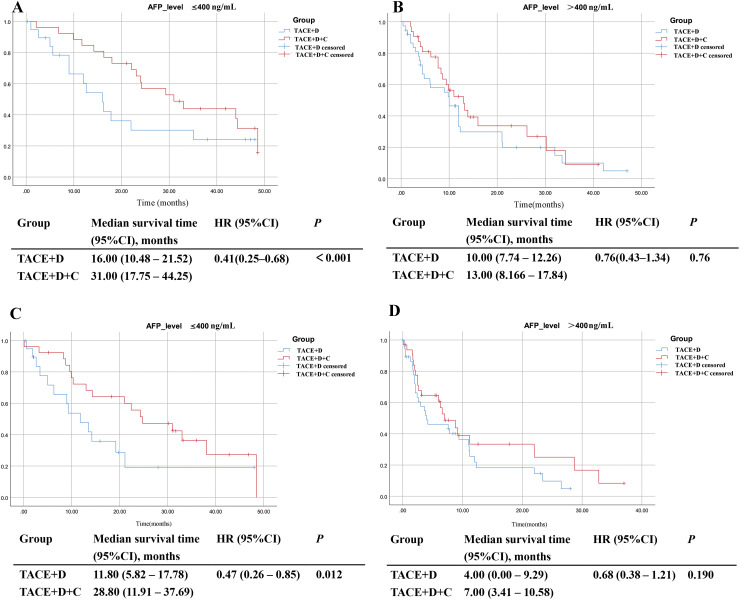
Subgroup analysis of OS **(A, B)** and PFS **(C, D)** according to AFP level. TACE, Transarterial Chemoembolization; D, donafenib; C, camrelizumab; CI, confidence interval; AFP, alpha-fetoprotein. HR, hazard ratio.

In the AFP ≤ 400 ng/mL subgroup, the TACE+D+C group achieved significantly longer mOS (31.00 months vs 16.00 months; *P* < 0.001) and mPFS (28.8 months vs 11.8 months; *P* = 0.012) compared with the TACE+D group (**Figure 8A, C**). In contrast, in the AFP > 400 ng/mL subgroup, no significant differences were observed between the TACE+D+C and TACE+D groups in terms of mOS (13.00 months vs 10.00 months; *P* = 0.760) or mPFS (7.00 months vs 4.00 months; *P* = 0.190) (**Figure 8B, D**).

#### Subgroup analysis stratified by PIVKA-II level

**Figure 9:**
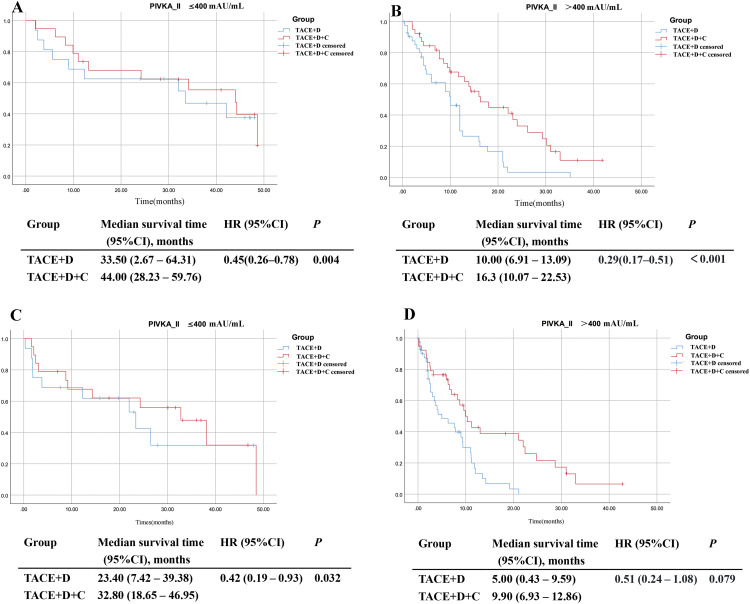
Subgroup analysis of OS **(A, B)** and PFS **(C, D)** according to PIVKA-II. TACE, Transarterial Chemoembolization; D, donafenib; C, camrelizumab; CI, confidence interval; PIVKA-II, Protein Induced by Vitamin K Absence or Antagonist-II; HR, hazard ratio.

In the PIVKA−II ≤ 400 mAU/mL subgroup, the TACE+D+C group exhibited significantly longer mOS (44.00 months vs 33.50 months; *P* = 0.004) and mPFS (32.8 months vs 23.4 months; *P* = 0.032) compared with the TACE+D group (**Figure 9A, C**). In the PIVKA−II > 400 mAU/mL subgroup, the TACE+D+C group achieved significantly longer mOS (16.30 months vs 10.00 months; *P* < 0.001) but only showed a numerically longer mPFS (9.90 months vs 5.00 months; *P* = 0.079) without reaching statistical significance compared with the TACE+D group (**Figure 9B, D**).

#### Subgroup analysis stratified by BCLC stage

**Figure 10:**
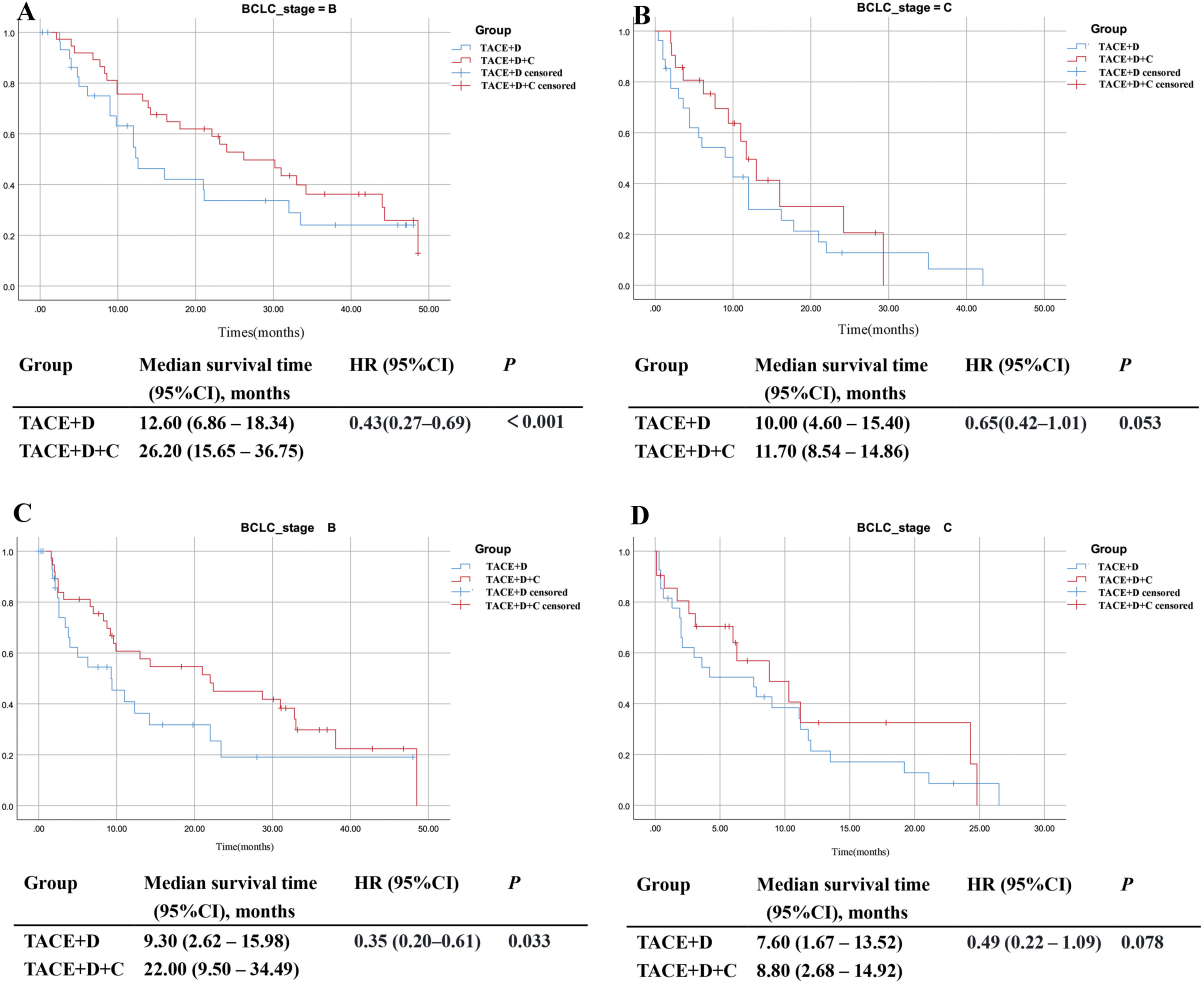
Subgroup analysis of OS **(A, B)** and PFS **(C, D)** according to BCLC stage. TACE, Transarterial Chemoembolization; D, donafenib; C, camrelizumab; CI, confidence interval; BCLC, Barcelona Clinic Liver Cancer; HR, hazard ratio.

In the BCLC B subgroup, the mOS in the TACE+D+C group was significantly longer than that in the TACE+D group (26.20 months vs 12.60 months; *P* < 0.001). The mPFS was also significantly longer in the TACE+D+C group than in the TACE+D group (22.00 months vs 9.30 months; *P* = 0.033) (**Figure 10A, C**). However, in the BCLC C subgroup, although the TACE+D+C group exhibited numerically longer mOS (11.7 months vs 10.00 months; *P* = 0.053) and mPFS (8.80 months vs 7.60 months; *P* = 0.078) compared with the TACE+D group, the differences did not reach statistical significance (**Figure 10B, D**).

### Safety

**Figure 11:**
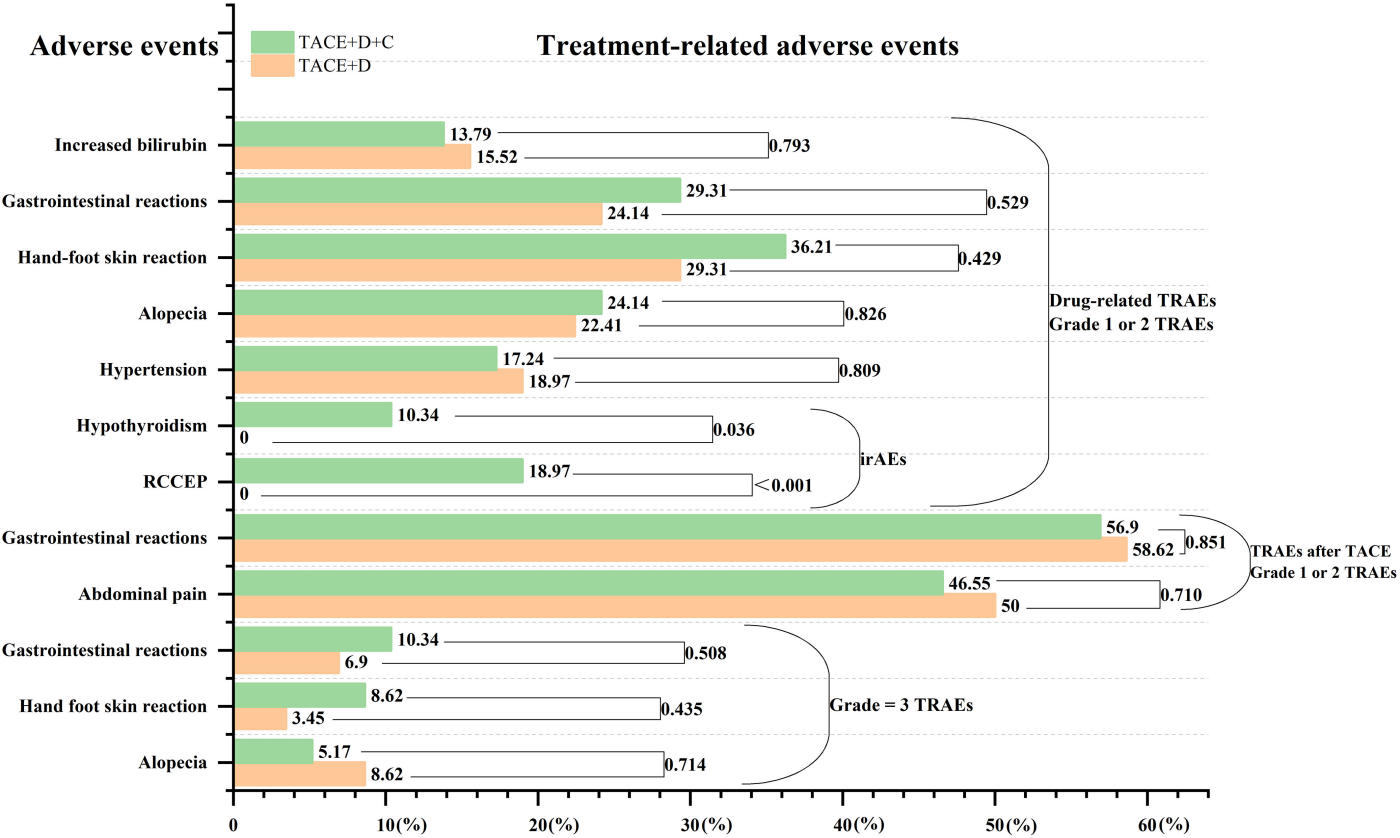
TRAEs between the two groups. Data are presented as numbers (percentages). RCCEP, reactive cutaneous capillary endothelial proliferation; TACE+D, Transarterial Chemoembolization combined with donafenib; TACE+D+C, Transarterial Chemoembolization combined with donafenib and camrelizumab; TRAEs, treatment-related adverse events; irAEs, immune-related adverse events.

After PSM, the safety profiles of the two groups were comparable (**Figure 11**), with no statistically significant differences in the incidences of drug-related TRAEs, TACE-associated TRAEs, and grade 3 TRAEs (all *P* > 0.05). In the TACE+D group, donafenib dose reduction occurred in 18.97% of patients; in the TACE+D+C group, donafenib and camrelizumab dose reductions occurred in 24.14% and 10.34% of patients, respectively. All dose reductions described above were attributable to AEs. Notably, irAEs were only observed in the TACE+D+C group. All irAEs were grade 1-2, including hypothyroidism (6/58, 10.34%) and RCCEP (11/58, 18.97%), with no grade ≥ 3 irAEs recorded in this group (**Supplementary Table 3****).**

For the clinical management of irAEs, patients with hypothyroidism received standardized levothyroxine replacement therapy, and those with RCCEP were administered glucocorticoid therapy as needed. For all TRAEs (including irAEs) in the final study population of both groups, the primary intervention strategies were symptomatic management and temporary treatment discontinuation when necessary; no patients required permanent treatment discontinuation due to AEs, nor did any patients or their family members request to withdraw from treatment due to AEs.

Throughout the entire study period, no grade ≥ 4 TRAEs were reported in either group, and there were no treatment-related deaths. All TRAEs were successfully resolved with the above-mentioned clinical interventions, and no persistent AEs or long-term sequelae observed during follow-up.

## Discussion

For uHCC, a combination of local and systemic treatment strategies has become the primary therapeutic approach. Specifically, the regimen of TACE plus targeted therapy and ICIs has been validated by the EMERALD-1 (24) and LEAP-012 (25) trials, demonstrating that TACE plus targeted therapy and ICIs significantly prolong OS and PFS in patients. However, it is important to note that both randomized controlled trials exhibited significant clinical enrollment limitations, failing to fully represent the characteristics of real-world uHCC patients. Specifically, EMERALD-1 only included 6%-8% of HCC patients with vascular invasion, while LEAP-012 explicitly excluded this subgroup. Vascular invasion, a core factor for poor prognosis in uHCC, occurs in 30%-50% of real-world cases (26). Furthermore, 98% and 89% of patients in the two studies, respectively, had Child-Pugh A grade, lacking efficacy data to support for patients with poor hepatic reserve (Child-Pugh B/C grade). In addition, both trials employed regimens that combined targeted agents with ICIs. Moreover, the study populations had a high proportion of cases with hepatitis C virus (HCV)-related and alcohol-associated HCC, which is inconsistent with the epidemiological characteristics of HCC in China, where HBV-related HCC predominates (27). Compounding these issues, the therapeutic agents used in these international trials pose substantial barriers to accessibility and affordability for domestic patients, creating a bottleneck for the translational application of their findings in routine Chinese clinical practice.

This study was a single-center retrospective analysis of patients with uHCC who received TACE combined with systemic therapy, and PSM was applied to minimize selection bias. A treatment assignment prediction model was constructed, demonstrating good discriminatory ability (AUC = 0.726, 95% CI: 0.65-0.79). Additionally, a Rosenbaum sensitivity analysis was conducted to assess the impact of unmeasured confounding factors on the results. At a gamma value of 1.6, the triple therapy regimen remained associated with a statistically significant treatment effect (upper limit *P*-value = 0.049), indicating that unobserved confounding factors would need to increase the odds ratio of treatment assignment by at least 1.6-fold to eliminate the significant association between the TACE+D+C group and survival benefit. These findings fully validate the robustness of the PSM results and mitigate the impact of potential residual confounders on the study conclusions. To further validate the stability of the results, a sensitivity analysis was conducted. Compared with the TACE+D group, the TACE+D+C group demonstrated statistically significant improvements in OS and PFS in both the unadjusted and fully adjusted models. These results further confirm that the survival benefit of the TACE+D+C group is independent of confounding factors, supporting the reliability and robustness of this triple therapy in patients with uHCC.

Our efficacy analysis results showed that before PSM, the TACE+D+C group had significantly longer mPFS (17.5 months vs 7.8 months, *P* = 0.003) and mOS (24.0 months vs 12.4 months, *P* = 0.023) than the TACE+D group. After PSM, the survival advantage of the triple combination regimen remained statistically significant and clinically meaningful: the mPFS of the TACE+D+C group was 13.0 months (vs 7.8 months in the TACE+D group, *P* = 0.007), and the mOS was 23.1 months (vs 12.0 months in the TACE+D group, *P* = 0.022). The positive findings of our study are consistent with the core conclusions of the EMERALD-1 and LEAP-012 trials, further validating the synergistic value of TACE combined with targeted therapy and ICIs, while highlighting distinct novelty and clinical relevance. Although the post-PSM mPFS (13.0 months) of the triple therapy in our study was lower than that in EMERALD-1 (15.0 months) and LEAP-012 (14.6 months), this difference can be reasonably explained by the inherent differences in patient characteristics. Specifically, our study included 34.48%-44.83% of patients with HCC who had portal vein invasion, as well as a significant proportion of patients with Child-Pugh Class B liver function. In previous studies, patients with portal vein invasion and poorer liver function have generally exhibited a more complex tumor microenvironment (characterized by more severe hypoxia and stronger immunosuppression) and have had poorer prognoses (28, 29). In contrast, the superior efficacy observed in EMERALD-1 and LEAP-012 relied on an ideal HCC population with low-risk features and well-preserved liver function reserve. This discrepancy precisely highlights the core value of our study: demonstrating significant survival benefit with the TACE+D+C group in a real-world population of uHCC patients with a high prevalence of poor prognostic features. Furthermore, our study included a high proportion of HBV-infected patients and employed the domestically produced donafenib combined with camrelizumab regimen. Both agents are included in China’s National Reimbursement Drug List, aligning with domestic epidemiological characteristics while substantially enhancing treatment accessibility and affordability (16, 17). Furthermore, the outcomes of this study were superior to those reported by Yang et al. (30) for the TACE+sorafenib+immunotherapy group, with an extension of 10.8 months in mOS (23.1 months vs 12.3 months) and 6.1 months in mPFS (13.0 months vs 6.9 months), which further corroborates the efficacy advantage of the TACE+D+C regimen.

To further verify the temporal stability of the therapeutic effect of the TACE+D+C group and eliminate the interference of time-dependent bias, we conducted milestone survival analyses at 6 and 12 months after treatment initiation, with strict inclusion criteria requiring patients to be progression-free and alive at the respective milestone time points. Milestone analysis is a robust method to reduce immortal time bias in observational studies of locoregional plus systemic combination therapy for HCC and has been increasingly adopted in high-level studies of TACE combined with targeted-immunotherapy regimens (31). The results showed that at the 6-month milestone, the mOS (20.20 months vs 10.20 months, *P* = 0.036) and mPFS (24.3 months vs 14.2 months, *P* = 0.027) of the TACE+D+C group were significantly longer than those of the TACE+D group. At the 12-month milestone, the survival advantage of the TACE+D+C group was more significant, with mOS of 21.00 months vs 9.10 months (*P* = 0.001) and mPFS of 21.00 months vs 10.00 months (*P* = 0.002). These findings confirm that the synergistic therapeutic effect of TACE, donafenib and camrelizumab is durable and long-lasting, rather than a transient tumor response. This is consistent with the biological rationale that TACE induced immunogenic cell death synergizes with anti-PD-1 agents and anti-angiogenics to establish sustained tumor control (32). Our results provide critical evidence supporting the sustained survival benefit of this triple regimen, with important clinical implications for patients who survive beyond 6–12 months of initial therapy, and further strengthen the real-world evidence base for long-term application.

Based on the independent prognostic factors identified by multivariate Cox regression analysis, we performed subgroup analyses stratified by AFP level, PIVKA-II, and BCLC stage to clarify the population applicability of the TACE+D+C group and provide an evidence base for personalized uHCC treatment. The results showed that in the subgroup of patients with AFP ≤ 400 ng/mL, the TACE+D+C group resulted in significantly prolonged mOS and mPFS compared with the TACE+D group. Conversely, among patients with AFP > 400 ng/mL, there were no significant intergroup differences in mOS or mPFS, implying that the clinical advantage of triple combination therapy is mainly confined to those with relatively low AFP concentrations. For PIVKA-II stratification, the TACE+D+C group showed superior therapeutic efficacy in both the ≤ 400 mAU/mL and > 400 mAU/mL subgroups, and the mOS of the low PIVKA-II subgroup reached 44.00 months, reflecting the prominent therapeutic effect of the triple combination regimen in patients with low tumor marker levels. These results align with recent evidence that tumor markers predict prognosis and treatment response in HCC, and extend findings to the donafenib plus camrelizumab backbone (33). In the BCLC-stratified analysis, the TACE+D+C group demonstrated significantly longer mOS and mPFS compared to the TACE+D group in BCLC B stage patients. Collectively, the subgroup analysis results further confirm that the TACE+D+C group has broad applicability, delivering survival benefits to uHCC patients across different tumor marker levels and BCLC stages. This provides more refined and targeted clinical guidance for selecting treatment strategies in uHCC.

We used ORR and DCR as secondary endpoints to quantitatively evaluate treatment efficacy in solid tumors. PSM analysis revealed that the PR in the TACE+D+C group was significantly higher than that in the TACE+D group (37.93% vs 20.69%, *P* = 0.041), while the ORR (62.07% vs 36.21%, *P* = 0.005) and DCR (86.21% vs 70.69%, *P* = 0.042) were both significantly higher than in the TACE+D group. These results are consistent with the findings of Li et al. (34) in a multicenter retrospective study, which reported that the TACE+D+C group achieved significantly higher ORR (50.6% vs 32.1%, *P* < 0.001) and DCR (89.2% vs 74.3%, *P* = 0.002) than the TACE+D group. The significant improvement in tumor response indicators fully reflects the enhanced tumor control ability of the triple combination regimen. The TACE+D+C group demonstrated superior efficacy in our study due to a triple synergistic mechanism involving local control, anti-angiogenesis, and immune activation. The combination of donafenib and camrelizumab was selected based on robust pharmacological and mechanistic rationale, not as a random pairing. First, TACE induces tumor cell apoptosis through catheter-based infusion of chemotherapeutic agents and simultaneously occludes the local tumor blood supply, resulting in tumor ischemia and necrosis. However, this ischemic necrosis releases substantial tumor-associated antigens (TAAs) and hypoxia-inducible factor-1α (HIF-1α), which in turn trigger the release of factors such as vascular endothelial growth factor (VEGF) and fibroblast growth factor (FGF), ultimately promoting tumor cell proliferation and neovascularization (35, 36). Donafenib, a novel molecularly targeted drug following sorafenib, inhibits multiple tyrosine kinases, Raf kinase, and downstream Raf/Mek/Erk signaling pathways, thereby suppressing tumor cell proliferation and angiogenesis; its combination with TACE further enhances tumor cell killing efficacy (11). Second, PD-L1 expression is upregulated after TACE, which inhibits T-cell activity and impairs the tumor-killing capacity of the immune system (37). As an anti-PD-1 monoclonal antibody, camrelizumab blocks the PD-1/PD-L1 interaction between tumor cells and T lymphocytes, reversing T-cell dysfunction, and restoring cytotoxic anti-tumor immune responses (38).

More importantly, the immune microenvironment remodeling effect of donafenib can directly potentiate the immune activation efficacy of camrelizumab. Preclinical studies have confirmed that donafenib can inhibit the secretion of immunosuppressive factors such as VEGF and IL-6, reduce the infiltration of regulatory T cells (Tregs) and myeloid-derived suppressor cells (MDSCs), decrease the levels of immunosuppressive cytokines, and simultaneously promote the recruitment of CD8+ cytotoxic T lymphocytes (CTLs) into tumor tissues, thereby creating favorable microenvironmental conditions for immunotherapy (39). Meanwhile, camrelizumab blocks the PD-1/PD-L1 pathway to reverse T-cell dysfunction. The activated CTL response can further eliminate donafenib-sensitive tumor cells, forming a closed-loop synergistic effect of anti-angiogenesis and immune activation (40).

This triple synergistic mechanism establishes the efficacy foundation for the TACE+D+C regimen. Moreover, compared with combinations of other targeted agents and ICIs, the pairing of donafenib and camrelizumab confers unique pharmacological advantages that further enhance the clinical application value of our study findings. With respect to the ICIs component, the Fab fragment of camrelizumab possesses a unique arginine triplet (Arg84/Arg86/Arg88) that forms a double-dentate hydrogen bond with the Asn66 and Gly68 residues on PD-1. This binding affinity is 3.2 times higher than that of pembrolizumab, with a 68% lower dissociation rate than nivolumab, prolonging the duration of immune synapse engagement (41). Flow cytometry analysis demonstrated significantly higher PD-1 receptor occupancy in hepatocellular carcinoma patients treated with camrelizumab compared to nivolumab (i 95% at 72 hours; means 98.2% ± 1.3% vs 85.4% ± 4.1%, *P* < 0.001). This enhanced target binding capacity yields superior immune activation effects, while its short half-life and high target affinity characteristics reduce the incidence of irAEs (42, 43). In terms of targeted drug advantages, the deuterated structure of donafenib inhibits the N-demethylation pathway, reducing the generation of toxic metabolites. The incidence of Grade 3 AEs was significantly lower than that of sorafenib (38% vs 50%, *P* = 0.018) (44, 45). Notably, the spectrum of donafenib-related AEs does not overlap with that of camrelizumab, which ensures the safety of combination therapy. Furthermore, real-world studies have confirmed that camrelizumab confers statistically significantly superior mOS and mPFS compared with pembrolizumab and nivolumab in patients with uHCC (46, 47). In contrast to sorafenib, donafenib also achieves statistically significant improvements in ORR (4.6% vs 2.7%, HR = 1.71, 95% CI: 1.06-2.76, *P* = 0.0288) and DCR (30.8% vs 28.7%, *P* = 0.0472) (10).

In terms of safety, all TRAEs in our study were Grade ≤ 3, with no Grade ≥ 4 or unexpected AEs reported. The overall incidence of AEs in the TACE+D+C group was not significantly different from that in the TACE+D group, confirming the favorable safety profile and good clinical tolerability of the triple combination regimen. This outcome is closely related to the pharmacological properties of donafenib and camrelizumab. The most common AEs in the TACE+D+C group were donafenib-related reactions, including hand-foot skin reactions (36.21%), gastrointestinal reactions (29.31%), and alopecia (24.14%), which were consistent with the safety data reported in previous studies involving donafenib-based regimens (10). However, the mechanisms underlying donafenib-induced hand-foot skin reactions, gastrointestinal reactions, and alopecia remain unclear: hand-foot skin reactions may relate to the drug’s effects on skin metabolism, angiogenesis, and immunomodulation (11); Gastrointestinal reactions are often attributed to prolonged drug residence time in the gastrointestinal tract, which stimulates the mucosa and causes diarrhea. Regarding the mechanism of donafenib-induced alopecia, emerging evidence suggests that as a molecular-targeted agent, donafenib not only inhibits tumor angiogenesis but also potentially impairs the activity of hair follicle melanocyte stem cells. Additionally, it suppresses multiple receptor tyrosine kinases, including the c-KIT signaling pathway, thereby inhibiting tyrosinase and related protein activity in melanocytes, hindering melanin synthesis and metabolism, compromising hair follicle health, and ultimately leading to alopecia (11, 48, 49).

Notably, characteristic AEs associated with camrelizumab were observed in this study, including RCCEP (18.97%) and hypothyroidism (10.34%). The specific mechanism by which camrelizumab induces RCCEP is still unclear. It is hypothesized that camrelizumab may temporarily inhibit the VEGF signaling pathway, which is mediated by vascular endothelial growth factor receptor 2 in endothelial cells, potentially resulting in endothelial injury (50). Concurrently, it excessively activates immune functions, leading to the upregulation of VEGF-A. This disruption creates an imbalance between proangiogenic and antiangiogenic factors in skin tissue, ultimately resulting in the reactive proliferation of capillary endothelial cells (44, 51). The exact molecular mechanisms underlying hypothyroidism caused by camrelizumab remain unclear. However, current studies predominantly indicate that camrelizumab leads to abnormal T-cell activation, which cross-recognizes thyroid autoantibodies and causes damage to thyroid follicles (52–54). Ying Chen et al. (55) discovered significant infiltration of CD163+ macrophages and thyroid follicle destruction in patients with immunosuppressant-induced thyroid dysfunction. The above research indirectly elucidates the mechanism behind the hypothyroidism triggered by camrelizumab. Despite existing research, there is a paucity of sufficient evidence regarding the specific mechanisms underlying the AEs caused by camrelizumab. In this study, the AEs demonstrated improvement following appropriate symptomatic treatment. Overall, the TACE+D+C regimen exhibits favorable safety characteristics; however, the specific mechanisms underlying its associated AEs require further investigation to inform the clinical prevention and management of AEs, as well as to guide the optimization of this treatment protocol.

This study has several inherent limitations that merit acknowledgment. First, the retrospective, real-world design is inherently associated with challenges in comprehensive data acquisition. Given the considerable heterogeneity of clinical treatment regimens for uHCC in routine practice, complete datasets encompassing multimodal imaging characteristics, serial longitudinal clinical follow-up records, detailed documentation of TRAEs, and long-term survival outcomes could not be obtained for all study participants. A notable limitation stemming from this retrospective design is the absence of systematically collected longitudinal data on irAEs. Without prospectively predefined, standardized time points for irAE assessment and follow-up, we were unable to precisely characterize the temporal dynamics of irAEs, including their onset time, peak incidence, and resolution course. This knowledge gap impedes an in-depth understanding of the safety profile of the TACE+D+C regimen, and more specifically, the time-dependent toxicological characteristics of the combination of anti-angiogenic therapy and ICIs—an issue critical to the clinical management of this regimen.

Second, the clinical application window of donafenib is relatively narrow, as it was approved for the first-line treatment of uHCC in 2021. This restricted the case accumulation period for the present study (January 2021 to January 2024), particularly in the early phase of donafenib’s clinical use when its combination with TACE and camrelizumab remained in the exploratory stage of clinical practice. Furthermore, the substantial inherent heterogeneity of uHCC management in real-world clinical practice, together with our stringent study inclusion criteria (e.g., receipt of the TACE+D+C or TACE+D regimen alone without subsequent surgical resection), further restricted the number of eligible patients, inevitably resulting in a relatively small final sample size. This sample size limitation, in turn, resulted in small subgroup sizes for stratified analyses; as a consequence, some endpoints (e.g., partial survival indicators in BCLC stage-stratified analyses) lacked sufficient statistical power to detect significant differences, which may have obscured potential treatment benefits in specific patient subsets.

Third, the optimal integration of immunotherapy into the multimodal treatment paradigm for uHCC remains to be refined. Although ICIs have become a core component of combination therapies for uHCC, their precise clinical deployment—including patient stratification and optimal utilization in specific subpopulations—remains poorly defined. While robust clinical evidence supports the use of ICI−based regimens in the second−line or later settings for uHCC, high−quality data validating their first−line application in high−risk subgroups (e.g., patients with vascular invasion or Child−Pugh class B/C liver function) are still scarce (56). Thus, future prospective studies should prioritize the identification and validation of predictive biomarkers to guide precise patient selection, clarify benefit stratification for ICI-based combination therapies, and expand the evidence-based clinical indications for such regimens in uHCC.

Fourth, the potential of the TACE+D+C regimen to facilitate conversion therapy for initially unresectable uHCC was not explored in the present study. Despite the promising survival outcomes observed with this triple combination therapy, the rate at which it can downstage advanced tumors to achieve resectability—a key clinical endpoint for improving curative potential in uHCC—remains unknown. This critical question could not be addressed due to the retrospective, single-center design and modest sample size of our analysis, which limited the ability to quantify conversion resection rates and assess their associated clinical outcomes.

Accordingly, large-scale, prospective, multicenter real-world studies are warranted to validate the findings of the present analysis and enhance the robustness and external generalizability of the clinical evidence for the TACE+D+C regimen. Such studies should incorporate prospectively designed, standardized irAEs monitoring protocols—including scheduled assessment time points (e.g., biweekly evaluations for the first 3 months of treatment, followed by monthly assessments thereafter)—to systematically capture longitudinal irAEs data, enabling precise characterization of their temporal dynamics and the optimization of safety management strategies for this combination regimen. Additionally, future investigations should focus on quantifying the conversion therapy potential of the TACE+D+C regimen, identifying predictive biomarkers for treatment response and toxicity, and defining the optimal role of this regimen in the first-line setting for high-risk uHCC subgroups, to further advance the precision and individualization of uHCC treatment.

## Conclusion

In conclusion, the present study demonstrates that the TACE+D+C regimen significantly improves ORR, PFS, and OS in uHCC patients compared with the TACE+D regimen, with a comparable safety profile and manageable TRAEs. The robust PSM design, coupled with milestone and subgroup analyses, confirms the durability and broad applicability of these therapeutic benefits. These findings support the TACE+D+C combination as a viable option for improving the prognosis of uHCC patients, particularly those with adverse prognostic features. However, the single-center retrospective nature of the study underscores the need for validation in large-scale prospective randomized controlled trials to confirm the generalizability of these conclusions.

## Data Availability

The original contributions presented in the study are included in the article/**Supplementary Material**. Further inquiries can be directed to the corresponding author.
