# Supplementary material for: Efficacy and safety of transarterial chemoembolization plus donafenib with or without camrelizumab for unresectable hepatocellular carcinoma: a propensity score matching analysis
Source: Front Immunol. 2026 Apr 16;17:1694973. doi: 10.3389/fimmu.2026.1694973 (PMC13128621; doi:10.3389/fimmu.2026.1694973)
Supplement: Supplementary file 1 [file DataSheet1.docx]

**Supplementary information**

Supplementary Figure 1 ROC curve for predicting receipt of specified treatment after incorporating covariates.

Supplementary Table 1 Results of Rosenbaum sensitivity analysis for overall survival according to different gamma values.

Supplementary Table 2 Treatment responses before PSM **(A)** and after PSM **(B)** for the two groups.

Supplementary Table 3 TRAEs between the two groups

**
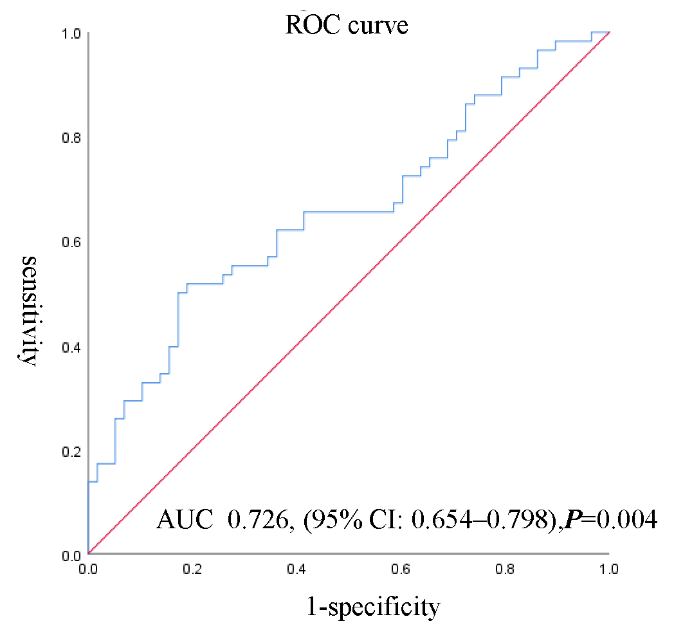
**Supplementary Figure 1 ROC curve for predicting receipt of specified treatment after incorporating covariates

Supplementary Table 1 Results of Rosenbaum sensitivity analysis for overall survival according to different gamma values.

| **Gamma** | ***P*_upper** |
| --- | --- |
| 1.0 | 0.001897 |
| 1.1 | 0.004562 |
| 1.2 | 0.009158 |
| 1.3 | 0.017231 |
| 1.4 | 0.023867 |
| 1.5 | 0.030125 |
| 1.6 | 0.049218 |
| 1.7 | 0.075826 |
| 1.8 | 0.083671 |
| 1.9 | 0.098268 |
| 2.0 | 1.273894 |

**Abbreviation:** P_upper, upper bound of P-value; Gamma, sensitivity parameter reflecting the strength of unmeasured confounding.

Supplementary Table 2 Treatment responses before PSM **(A)** and after PSM **(B)** for the two groups.

| Tumor response, n (%) | Before PSM | | | After PSM | | |  |
| --- | --- | --- | --- | --- | --- | --- | --- |
|  | TACE+D group(n=119) | TACE+D+C group(n=67) | *P-*value | TACE+D group(n=58) | TACE+D+C group(n=58) | *P-*value | |
| CR | 13 (10.92) | 16 (23.88) | 0.019 | 9 (15.52) | 14 (24.14) | 0.244 | |
| PR | 29 (24.37) | 26 (38.81) | 0.038 | 12 (20.69) | 22 (37.93) | 0.041 | |
| SD | 47 (39.50) | 15 (22.39) | 0.017 | 20 (34.48) | 14 (24.14) | 0.221 | |
| PD | 30 (25.21) | 10 (14.92) | 0.101 | 17 (29.31) | 8 (13.79) | 0.042 | |
| ORR | 42 (35.29) | 42 (62.69) | <.001 | 21 (36.21) | 36 (62.07) | 0.005 | |
| DCR | 89 (74.79) | 57 (85.08) | 0.101 | 41 (70.69) | 50 (86.21) | 0.042 | |

Abbreviations: TACE+D, Transarterial Chemoembolization combined with donafenib; TACE+D+C, Transarterial Chemoembolization combined with donafenib and camrelizumab; CR, complete response; PR, partial response; SD, stable disease; PD, progressive disease; ORR, objective response rate; DCR, disease control rate

Supplementary Table 3 TRAEs between the two groups

| Adverse events, n (%) | **Grade 1or 2 TRAEs** | | | **Grade 3 TRAEs** | | |
| --- | --- | --- | --- | --- | --- | --- |
|  | TACE+D group(n=58) | TACE+D+C group(n=58) | *P-*value | TACE+D group(n=58) | TACE+D+C group(n=58) | *P-*value |
| **Drug-related TRAEs** |  |  |  |  |  |  |
| Increased bilirubin | 9 (15.52) | 8 (13.79) | 0.793 | 0 | 0 | 0 |
| Gastrointestinal reactions | 14 (24.14) | 17 (29.31) | 0.529 | 4 (6.90) | 6 (10.34) | 0.508 |
| Hand-foot skin reaction | 17 (29.31) | 21 (36.21) | 0.429 | 2 (3.45) | 5 (8.62) | 0.435 |
| Hypertension | 11 (18.97) | 10 (17.24) | 0.809 | 0 | 0 | 0 |
| Alopecia | 13 (22.41) | 14 (24.14) | 0.826 | 5 (8.62) | 3 (5.17) | 0.714 |
| **irAEs** |  |  |  |  |  |  |
| Hypothyroidism | 0 (0.00) | 6 (10.34) | 0.036 | 0 | 0 | 0 |
| RCCEP | 0 (0.00) | 11 (18.97) | <0.001 | 0 | 0 | 0 |
| **TRAEs after TACE** |  |  |  |  |  |  |
| Gastrointestinal reactions | 34 (58.62) | 33 (56.90) | 0.851 | 0 | 0 | 0 |
| Abdominal pain | 29 (50.00) | 27 (46.55) | 0.710 | 0 | 0 | 0 |

Abbreviations: RCCEP, reactive cutaneous capillary endothelial proliferation; TACE+D, Transarterial Chemoembolization combined with donafenib; TACE+D+C, Transarterial Chemoembolization combined with donafenib and camrelizumab; irAEs, immune-related adverse events
